# Subclinical Aortic Insufficiency Causing Cardiogenic Shock Years After Impella Placement

**DOI:** 10.7759/cureus.43699

**Published:** 2023-08-18

**Authors:** Ali Syed, Lucian Durham, Joshua Melamed, Paul J Pearson

**Affiliations:** 1 School of Medicine, Medical College of Wisconsin, Milwaukee, USA; 2 Cardiac Surgery, Medical College of Wisconsin, Milwaukee, USA; 3 Thoracic Surgery, Medical College of Wisconsin, Milwaukee, USA

**Keywords:** aortic valve replacement, thyroid storm, aortic valve insufficiency, cardiogenic shock, impella

## Abstract

A 28-year-old male with atrial fibrillation and thyrotoxicosis-induced heart failure underwent multiple interventions, including extracorporeal membrane oxygenation (ECMO), multiple valve repair/replacement, and Impella placement/removal. However, after a period of three years, the patient developed progressive aortic insufficiency (AI), which was attributed to damage caused by the prolonged use of the Impella device. The discussion highlights the importance of adhering to manufacturer guidelines for device use and emphasizes the need for careful examination during placement to minimize potential complications.

## Introduction

Left ventricular assist devices (LVADs) are mechanical pumps designed to support the failing left ventricle by augmenting cardiac output and improving systemic circulation [1]. These devices have revolutionized the management of end-stage heart failure, offering a bridge to heart transplantation or serving as a destination therapy for patients ineligible for transplantation [1,2]. With continuous technological advancements, LVADs now offer improved durability, enhanced patient survival, and better quality of life. Here we present a case where LVAD use caused aortic insufficiency (AI) three years after removal.

## Case presentation

A 28-year-old male with no prior medical history presented at an outside hospital for acute shortness of breath. The patient was found to be in atrial fibrillation (AF) with a rapid ventricular response. Electrical and chemical cardioversion were attempted to no avail. Further workup revealed thyrotoxicosis (thyroid stimulating hormone (TSH): 0.009, free T4: 4.64) as the cause for his first documented episode of AF. After three days, his left ventricular ejection fraction was found to be 13%, and he was transferred to our facility.

Upon arrival, he remained in AF with biventricular heart failure, worsening liver failure, renal failure, and sepsis, likely secondary to thyrotoxicosis. An Impella 5.0 LVAD was placed, and the patient was started on milrinone [3]. Milrinone was initiated at 11.875 mcg/min and titrated up to 47.5 mcg/min. Cardiac catheterization to sample tissue for giant cell myocarditis was negative. During catheterization, severe eccentric mitral regurgitation was noted, likely due to a flail segment of the anterior leaflet with a regurgitant jet pointed posteriorly. Left ventricular internal dimensions in diastole (LVIDd) and systole (LVIDs) measured 5.7 and 5.2 cm, respectively, and AI was absent. After 7 days, the patient's condition further stabilized, and a thyroidectomy was performed to address the thyroid storm. Over the next seven days, worsening hepatorenal failure ensued.

After 10 days, the patient's condition improved, leading to procedures that included Impella removal, mitral valve replacement, and tricuspid repair. However, attempts to wean him from the cardiopulmonary bypass circuit were unsuccessful, resulting in his placement on central extracorporeal membrane oxygenation (ECMO). His course was complicated by hemodynamic instability, necessitating prolonged intubation and multiple chest washouts. After five days, the decision was made to transition the patient once again from ECMO to Impella 5.0 support. At this point, the patient was noted to have "well-seated" mechanical tricuspid and mitral valves, as well as an appropriately angled Impella directed towards the left ventricular (LV) apex. The patient's condition steadily improved, and after 12 days, he was successfully weaned off the Impella [3].

**Figure 1:**
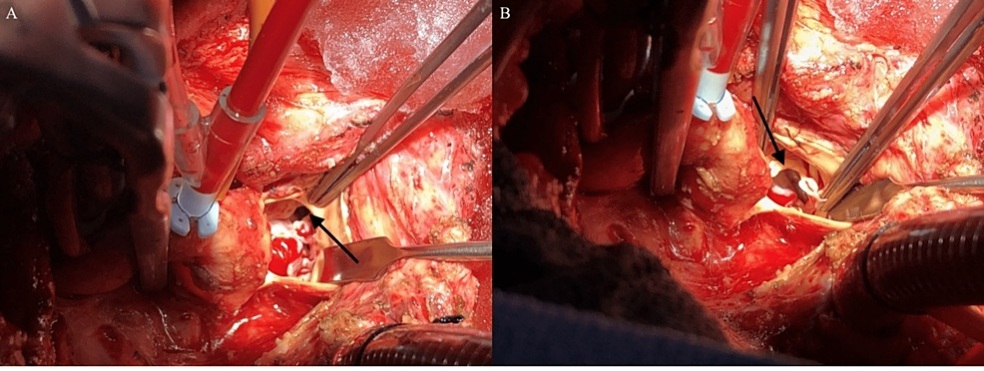
(A) An intraoperative image with an arrow pointing to the damage present in the right coronary cusp. (B) An intraoperative image with an arrow pointing at the indentation of the Impella into the right coronary cusp.

The patient spent 29 days on the floor and was discharged to rehabilitation shortly after, with moderate AI noted on discharge echocardiography (ECHO). Subsequent echocardiograms over the next two years consistently showed moderate AI. Cardiac magnetic resonance imaging 2.5 years later revealed severe AI but with an improving ejection fraction of 45%. Nonetheless, the patient was referred for surgical aortic valve replacement (AVR). Unfortunately, during this time, the patient presented to our hospital with symptoms of shortness of breath, dizziness, and diaphoresis. Further investigation revealed cardiogenic shock without arrhythmia, attributed to AI, nearly three years after his initial operation. He was taken to the operating room and underwent a re-do sternotomy and AVR. In the operating room, a fibrotic indentation was observed on the right cusp of the aortic valve, with a diameter similar to that of an Impella device (Figures 1A, 1B). The valve was excised and a 23 mm St. Jude mechanical valve was successfully implanted. His postoperative course was uneventful.

## Discussion

Our case is a unique experience of AI that developed over three years after the patient’s last Impella run. We theorize that placement of the Impella for a combined total of 27 days led to damage of the right-coronary cusp of the aortic valve, causing it to slightly mold around the Impella catheter. The damage to the cusp led to fibrosis with subsequent retraction. We believe that worsening fibrosis and retraction of the cusp caused progressive damage from the increase in the pressure of the eccentric jet back into the left ventricle, which in turn exacerbated the aortic regurgitation [4]. It is important to note that although AI can cause ventricular hypertrophy, dilation, and subsequent root enlargement leading to worsening insufficiency, this did not happen in our case.

Furthermore, Impella 5.0 was approved for up to six days of mechanical circulatory support at the time this case occurred [5]. While we exceeded the recommended duration of device usage, the critical condition of our patient warranted prolonged support. Nonetheless, due to the complications arising from extended use, we advocate adherence to the manufacturer's suggested usage guidelines whenever feasible. Additionally, thorough scrutiny using transesophageal echocardiography should be performed during placement to ensure minimal torque on the device. Torque can cause cusp tenting, likely leading to the complications we encountered.

## Conclusions

Prolonged use of mechanical circulatory support is a difficult decision. Our case highlights the difficult decision made by our providers with respect to our clinical scenario. The importance of post-operative trans-esophageal echocardiography can not be overstated to try and minimize torque which can cause the cusp of the aortic valve leaflets to tent open. Finally, if patients are noted to have mild to moderate aortic regurgitation post-LVAD usage, this case report provides reason to follow up closely and ensure intervention before a critical stage is reached. 
